# Molecular characterization of quinolone resistant *Shigella* spp. isolates from patients in Ardabil, Iran

**Published:** 2019-12

**Authors:** Roghayeh Teimourpour, Behnam Babapour, Majid Esmaelizad, Mohsen Arzanlou, Hadi Peeri-Doghaheh

**Affiliations:** 1 Department of Microbiology, School of Medicine, Ardabil University of Medical Sciences, Ardabil, Iran; 2 Microbiology Laboratory, Bu-Ali Hospital, Ardabil University of Medical Sciences, Ardabil, Iran; 3 Department of Biotechnology, Central Laboratory, Razi Vaccine and Serum Research Institute, Karaj, Iran

**Keywords:** *Shigella*, Fluoroquinolones, Nalidixic acid, Mutation, Gene

## Abstract

**Background and Objectives:**

*Shigella* is an etiological agent of shigellosis. Antibiotic therapy has a critical role in decreasing serious complications of shigellosis. The present study aimed to determine the multi-drug resistance strains and to detect fluoroquinolone related mutations.

**Materials and Methods:**

In this descriptive, cross sectional study, a total of 113 *Shigella* isolates were collected from 1280 patients admitted to Bu-Ali hospital in Ardabil province during 2015–17. Antibiotic resistance pattern of isolates was evaluated using Kirby Bauer method and finally, the MICs of ciprofloxacin were determined. In order to determine any mutations in QRDR region, *parC* and *gyrA* genes of resistant strains were amplified and sequenced.

**Results:**

*Shigella* spp. isolates were identified using *ipaH* amplification and *rfc* and *wbgz* genes were used for molecular detection of *S. flexneri* and *S. soneii*, respectively. Our results showed that the predominant species in Ardabil province was *S. sonnei* (69.91%). Most of isolates (82%) were resistant to trimethoprim/sulfamethoxazole (TMP/SMX); 51% were nalidixic acid resistant and 4.4% were floroquinolones resistant. All examined isolates were susceptible to imipenem (100%). Mutation in *gyrA* and *parC* genes were detected in all fluoroquinolone resistant isolates (5 isolates). Although, in this study the rate of resistance to ciprofloxacin was low, but in the lack of preventive strategy it will be a major challenge of public health in future.

**Conclusion:**

This study provided information on the prevalence and antimicrobial susceptibility patterns of *Shigella* isolates in Ardabil province, Iran. Also this study showed a high-level of resistance to commonly used antibiotics among *Shigella* isolates.

## INTRODUCTION

*Shigella*, a causative agent of bloody diarrhea, is a Gram-negative, non-motile bacillus that belongs to *Enterobacteriacae* family. *Shigella* genus including *S. dysenteriae, S. flexneri, S. boydii* and *S. sonnei* belong to A, B, C and D, serogroups, respectively. Shigellosis is a bacillary dysentery or invasive diarrhea that clinically manifested by watery or bloody diarrhea. This infection mostly transmitted through contaminated food and water. The disease caused by *S. dysenteriae* is very severe with high mortality and transmission rate that mostly predominant in developing countries (1, 2). In 2016, WHO estimated that about 165 million shigellosis occur each year worldwide (3). Malnutrition along with poor sanitation is the most important risk factors for increasing incidence of the disease and raising the mortality rate. Epidemiological studies around world indicated that *Shigella* spp. is one of the six leading pathogen attributed to life threatening in children. Therefore, early diagnosis and appropriate treatment of the disease plays a critical role in reducing the mortality rate. In the lack of effective antimicrobial treatment, the risk onset of serious complications, the duration of illness and death dramatically increased. According to literature guidelines fluoroquinolones especially ciprofloxacin is the first line of treatment. For multidrug resistance strain, pivmecillinam (amdinocillin pivoxil) and ceftriaxone are recommended. Azithromycin is the second-line therapy which is only prescribed for adult and is not recommended for children due to limited efficacy (4). Fluoroquinolone resistance in *Shigella* is correlated with multiple mechanisms including several mutations in quinolone resistance-determining region (QRDR), efflux pump and plasmid-mediated quinolone resistance (PMQR). QRDR encoding DNA gyrase (*gyrA* and *gyrB* genes) and topoisomerase IV (*parC* and *parE* genes) which among different resistance mechanisms has a predominant role. Presence of any mutation in these regions decrease binding of the drug to the enzyme-DNA complex. In fact, fluoroquinolones target two substantial bacterial enzymes including DNA gyrase and DNA topoisomerase IV which participate in DNA amplification. Alteration or single amino acid changes in either of these enzymes leads to fluoroquinolone resistance. Amino acids located in GyrA and ParC subunits are the most important amino acids which are covalently bound to DNA in both enzymes (5). Therefore, mutation in these amino acids is directly related to resistance to fluoroquinolones.

Identification of drug-resistant strains and their antibiotic resistance profile provide appropriate empirical treatment that will help in better control of the infection in the region of the study (6, 7). In this regard, in the present study, 113 isolates which was collected from Bu-Ali hospital during 2015–2017 were analyzed and the presence of any mutations in *gyrA* and *parC* genes were determined (8).

## MATERIALS AND METHODS

### Media and chemicals.

For isolation of *Shigella* spp. different media such as selenite F broth, Hektoen enteric agar (HE), Xylose lysine deoxycholate agar (XLD) were used (Himedia laboratories, Mumbai, India). For PCR reaction, oligonucleotide primers were synthesized by Bioneer Company (Daejon, South Korea). Ciprofloxacin powders were purchased from Bio Basic Company (Bio Basic Inc. Canada) for Minimum inhibitory concentration (MIC) determination. The antibiotic discs were prepared from Padtanteb Company (Padtanteb, Iran) and species specific antisera were purchased from SIFIN (Sifin diagnostics gmbh, Germany).

### Bacterial isolates.

In this descriptive, cross sectional study, from 2015 to 2017, a total of 113 *Shigella* isolates were collected from infected patients with <10 years old at Bu Ali hospital in Ardabil province. All isolates were examined by microbiological tests such as Gram staining, colony morphology on HE, XLD and EMB media and also IMViC tests (9). Serotyping of all biochemical positive isolates were done using *Shigella* specific antiserum. Whole bacterial genome was extracted using boiling method as described previously (10). Also molecular confirmation of *Shigella* spp, *S. flexneri* and *S. sonnei* were performed using amplification of *ipaH, rfc* and *wbgz* genes, respectively (Table 1). PCR reaction containing 10 pmol of each primer, MgCl_2_ 1.5 mM, 0.2 mM each dNTP, 1U Taq DNA polythermase was prepared at the total volume of 20 μl. Thermocycler program for PCR reaction was initial denaturation at 94°C for 5 min, followed by 30 cycles; denaturation at 94°C for 1 min, annealing at 60°C for 1 min, elongation at 72°C for 3 min. results of PCR reaction were analyzed via gel electrophoresis (10, 11).

**Table 1.** List of specific primers were used for amplification of *ipaH, rfc, wbgz* genes.

**Gene name**	**Primer sequence**	**PCR product size**	**Annealing time°C**
*ipaH*	F 5′ GTTCCTTGACCGCCTTTCCGATACCGTC3′ R 5′ GCCGGTCAGCCACCCTCTGAGAGTAC3′	619 bp (15)	60
*rfc*	F 5′ TTTATGGCTTCTTTGTCGG3′ R 5′ CTGCGTGATCCGACCATG3′	537 bp (16)	60
*wbgz*	F 5′ TCTGAATATGCCCTCTACGCT3′ R 5′GACAGAGCCCGAAGAACCG3′	430 bp (16)	60
*gyrA*	F 5′ TACACCGGTCAACATTGA GG3′ R 5′ TTA ATGATTGCCGCCGTCGG3′	648 (17)	52
*parC*	F 5′ GTCTGA ACT GGGCCTGAATGC3′ R 5′ AGCAGCTCGGAATATTTCGACAA3′	249 (17)	60

### Antimicrobial susceptibility testing.

General susceptibility test was done for ciprofloxacin (5 μg), nalidixic acid (30 μg), norfloxacin (10 μg), peroxazine (5 μg), gentamicin (10 μg), ceftriaxone (30 μg), cotrimoxazole (30 μg), impenamine (30 μg), amikacin (30 μg), and azithromycin (15 μg) using the disk diffusion method. *E. coli* ATCC-25922 and *S. flexneri* ATCC-12022 were used as controls. Minimum inhibitory concentration (MIC) for ciprofloxacin were determined for all identified isolates using agar dilution method (concentration rang: 0.25–256 μg/ml) (12).

### PCR amplification and sequencing.

The QRDRs of the *gyrA* and *parC* genes in clinical isolates were amplified using PCR technique and specific primers which are listed in Table 1. Extraction of genomic DNA from identified colonies was performed using boiling method (10). For all amplifications, a PCR mixture contains 1× PCR buffer, 1.5 mmol MgCl_2_, 0.2 mM of each dNTP, 1U of Taq DNA polymerase, 10 pmol of each primer, 1 μl of template DNA (0.5 μg), and sterile distilled water up to 25 μl were used. DNA amplification was done in the thermocycler PCR instrument (Bio Rad, USA). The temperature profile for *parC* and *gyrA* genes consisted of 95°C for 1 min; followed by 35 cycles denaturation at 95°C for of 30 sec, 30 sec of annealing at 52°C for *gyrA* and 60°C for *parC*, and 1 min of extension at 72°C and 10 min at 72°C for final extension. PCR products were analyzed using gel electrophoresis (1% agarose gel and 0.5/l mg ethidium bromide) UV transilluminator (10, 11, 13, 14).

PCR products of *gyrA* and *parC* genes related to five fluoroquinolone resistant strains were sequenced (Macrogene, South Korea) and acquired results were analyzed by DNAMAN, Chromas softwares and NCBI database (https://blast.ncbi.nlm.nih.gov/Blast.cgi) softwares.

### Statistical analysis.

Resistance patterns andstatistical comparisons were analyzed using SPSS software (version 20) and one-way ANOVA method.

## RESULTS

In the present study, a total of 113 *Shigella* isolates were recovered from patients with bacillary dysentery admitted to Bu Ali hospital during 2015–17. Of them, 79 cases (69.9%) were *Shigella sonnei*, 22 cases (19.4%) were *Shigella flexneri*, 9 cases (9.9%), *Shigella boydii* and 3 cases (6.2%) were *Shigella dysenteriae*. It was revealed that the dominant species in the Ardabil area is *Shigella sonnei*. Antimicrobial susceptibility test showed that 82% and 50.4% of isolates were resistant to co-trimoxazole and nalidixic acid respectively, but all (100%) were susceptible to imipenem. The rate of resistance to fluoroquinolones including ciprofloxacin, norfloxacin, ofloxacin was 4.4% (Table 2). Of the 113 identified strains, only 5 cases (4.4%) were resistant to ciprofloxacin. The MIC values for ciprofloxacin were determined for all identified strains that were in the range of 0.25 to 256 μg/ml. The MIC of ciprofloxacin against 90 isolates was less than 0.25 μg / ml, for 12 isolates was 0.5 μg/ml, 6 isolates was 1 μg/ml. Five isolates showed high level of resistance to ciprofloxacin. MIC for 3 cases (*Shigella sonnei*) was 16 μg/ml, 1 case (*Shigella boydii*) was 32 μg/ml and 1 case (*Shigella boydii*) 128 μg/ml. To determine any changes in the structure of DNA gyrase and topoisomerase IV enzymes, the QRDRs of corresponding genes (*gyrA* and *parC*) were amplified and sequenced.

**Table 2.** Resistance pattern of *Shigella* isolates collected from clinical specimens of Bu Ali Hospital in Ardabil, Iran

**Antibiotics**	***Shigella*** **spp. N= 113 N (%)**		

	**Resistance**	**Intermediate**	**Susceptible**
Ciprofloxacin	5 (4.4%)	-	108 (95.6%)
Ofloxacin	5 (4.4%)	-	108 (95.6%)
Norfloxacin	5 (4.4%)	-	108 (95.6%)
Nalidixic acid	57 (50.4%)	6 (5.4%)	50 (44.2%)
Cotrimoxazole	93 (82%)	5 (4.7%)	15 (13.3%)
Imipenem	-	-	113 (100%)
Amikacin	15 (13.3%)	2 (1.7%)	96 (85%)
Gentamicin	22 (19.4%)	5 (4.4%)	86 (76.2%)
Ceftriaxone	33 (29%)	6 (5%)	74 (66%)
Azithromycin	16 (14.2%)	10 (8.8%)	87 (77%)

Nucleotide sequence of DNA gyrase subunit A (accession no. ARS04283.1) and DNA topoisomerase IV subunit A (WP_001281839.1) were used for protein alignment.

In the case of *gyrA* gene, sequence analysis revealed that all five ciprofloxacin resistant isolates (100%) had Ser83Leu, Asp87Gly/Asn and Pro213Arg substitution. Glu210Gln only was seen in *Shigella sonnei* resistant strains. For the *parC* gene, all five ciprofloxacin resistant isolates carried Ser80Ile mutation while Tyr128Phe and Ser129Pro substitutions were detected in *Shigella boydii* resistant strains (Table 3).

**Table 3.** Alterations in *gyrA* and *parC* in clinical *Shigella* isolates

**No. of strains**	MICs (μg/ml) CIP*	**Nucleotide and amino acid change**						

		***gyrA* position**				***parC* position**		
	
		**Ser83 (TCG)**	**Asp87 (GAC)**	**Glu210 (GAA)**	**Pro213 (CCG)**	**Ser80 (AGC)**	**Tyr128 (TAT)**	**Ser129 (TCC)**
9 *(S. boydii)*	128	Leu (TTG)	Asn (AAC)	-	Arg (CGG)	Ile (ATC)	-	-
21 *(S.boydii)*	32	Leu (TTG)	Asn (AAC)	-	Arg (CGG)	Ile (ATC)	-	-
47 *(S. sonnei)*	16	Leu (TTG)	Gly (GGC)	Gln (CAA)	Arg (CGG)	Ile (ATC)	-	-
63 *(S. sonnei)*	16	Leu (TTG)	Gly (GGC)	Gln (CAA)	Arg (CGG)	Ile (ATC)	Phe (TTT)	PRO (CCC)
68 *(S. sonnei)*	16	Leu (TTG)	Gly (GGC)	Gln (CAA)	Arg (CGG)	Ile (ATC)	Phe (TTT)	PRO (CCC)

* ciprofloxacin

## DISCUSSION

In 2016, *Shigella* was the second leading cause of diarrhea-related deaths (18). Among different species of *Shigella*, *S. dysenteriae* is more severe and is more associated with other life-threatening complications such as haemolytic-uraemic syndrome (19). based on previous reports along with our study, this species is rare in Iran (20).

In contrast to other studies which indicated that *S. flexneri* is predominant species in developing country, the current study indicated that *S. sonnei* is the most common causative agent of shigellosis in Ardabil region which has been consisted with other studies (21). Also, in another study in Gorgan, *S. sonnei* was reported as a dominant species. Actually, the proximity of these two regions to each other may be the reason for this similarity. However, in the southern regions, the prevalence of *S. flexneri* is higher than other parts. In fact, the geographical regions, socio economic condition and personal hygiene affect the prevalence of *Shigella* species (22, 23).

In this study the highest rate of antibiotic resistance was related to co-trimoxazole (93%) which indicated that this antibiotic is not suitable for treatment of shigellosis. This rate was in accordance with the previous studies from Iran (21, 24). Although co-trimoxazole has been used extensively in the treatment of diarrhea, but overuse of it has led to a high degree of resistance.

The rate of resistance to nalidixic acid (57%) was relatively high in compare with previous dtudy (25). Therefore, it can be concluded that the administration of co-trimoxazole and nalidixic acid should be limited. Also it should be noted that the results of previous studies indicated that resistance to nalidixic acid was on the rise in Iran (21, 26, 27) which is in agreement with our result. Although fluoroquinolones and azithromycin are effective drugs, but decreased susceptibility to azithromycin have been described by Centers for Disease Control and Prevention (CDC) (1). Therefore surveillance of antibiotic resistance and appropriate use of available antibiotic are recommended.

Ciprofloxacin resistance rate (4.4%) of our study was close to a similar study in Tabriz (4.2%), located in proximity of Ardabil. In the present work, 5 isolates had high MIC for ciprofloxacin, of them, two isolates were *S. boydii* and the rests were *S. sonnei.* The MIC value in *S. boydii* was significantly higher than *S. sonnei* (Table 3). Although currently, these isolates are very rare but they could be pose as a serious concern in future.

According to previous studies, it is accepted that two or more mutations in both *gyrA* and *parC* subunits, especially at the highly conserved amino acids (Ser-83 and Asp-87) of *gyrA*, changes the structure of the DNA gyrase and DNA topoisomerase IV which are necessary to obtain moderate to high-level of resistance to ciprofloxacin (18, 28). Our sequencing results revealed that *Shigella* isolates with MIC greater than 16 μg/mL carried at least four mutations in *gyrA* (83, 87, 210, 213 codon) and *parC* (80, 128, 129 codon) genes. These results indicate that all of these changes are related to point mutations that resulted in substitution of several amino acids at GyrA and ParC subunits which ultimately reduced drug affinity to its target and high-level fluoroquinolone resistance (29). However, in one isolate with the highest MIC (128 μg/mL), other resistance mechanism such as efflux pump may be involved that needs to be investigated. In a study conducted by Ranjbar in Tehran, in two nalidixic acid-resistant *Shigella* strains, only one Ser83Leu mutation was observed (30). In another study, Yaghoubi et al. reported D87Y mutation that was not found in our work (31). However, they showed all ciprofloxacin-resistant isolates (MIC>32 mg/ml) had mutation in *gyrA* and *parC* genes at positions Ser83Leu/Asp87Gly and Ser80Ile respectively. In this study the most common mutations in *gyrA* and *parC* genes were Ser83Leu and Ser80Ile, relatively as seen in previous study in China (32). To our knowledge, other mutations including Glu210Gln and Pro213Arg substitutions are unique in the present work. The hallmark of the present study was to demonstrate the correlation of high-level of resistance to ciprofloxacin and rising MIC and several mutations in *gyrA* and *parC* genes during 2015–2017. Research over the past few years have shown that *Shigella* spp. have become resistant to formerly effective drugs and misuse of potent available antibiotics such as fluoroquinolones may repeat this scenario not so long ago (33, 34).

In conclusion, this study described fluoroquinolone resistance among *Shigella* spp. isolates collected from Ardabil province of Iran in 2015–2017. Our results provides valuable information in antibiotic resistant pattern of indigenous strains and suggest that acquisition of mutations in *gyrA* and *parC* genes plays a critical role in the development of high-level resistance to fluoroquinolones.

## CONCLUSION

This study provides baseline information on the prevalence of *Shigella* species on Ardabil province, antibiotic resistance pattern of isolates and association of the fluoroquinolone resistance with different mutations in QRDR region. These data will helpful in elucidating molecular mechanisms of drug resistance, development of new strategies to control the emergence and spread of MDR strain and providing appropriate empirical therapy against *Shigella* infections.
